# Examining the Usage, User Experience, and Perceived Impact of an Internet-Based Cognitive Behavioral Therapy Program for Adolescents With Anxiety: Randomized Controlled Trial

**DOI:** 10.2196/15795

**Published:** 2020-02-05

**Authors:** Ashley D Radomski, Alexa Bagnell, Sarah Curtis, Lisa Hartling, Amanda S Newton

**Affiliations:** 1 Department of Pediatrics University of Alberta Edmonton, AB Canada; 2 Department of Psychiatry Dalhousie University Halifax, NS Canada; 3 Department of Psychiatry Izaak Walton Killam Health Centre Halifax, NS Canada

**Keywords:** internet, cognitive behavioral therapy, computer-assisted therapy, anxiety, adolescents, clinical effectiveness, satisfaction, minimal clinically important difference, treatment adherence

## Abstract

**Background:**

Internet-based cognitive behavioral therapy (iCBT) increases treatment access for adolescents with anxiety; however, completion rates of iCBT programs are typically low. Understanding adolescents’ experiences with iCBT, what program features and changes in anxiety (minimal clinically important difference [MCID]) are important to them, may help explain and improve iCBT program use and impact.

**Objective:**

Within a randomized controlled trial comparing a six-session iCBT program for adolescent anxiety, *Being Real, Easing Anxiety: Tools Helping Electronically* (*Breathe*), with anxiety-based resource webpages, we aimed to (1) describe intervention use among adolescents allocated to *Breathe* or webpages and those who completed postintervention assessments (*Breathe* or webpage respondents); (2) describe and compare user experiences between groups; and (3) calculate an MCID for anxiety and explore relationships between iCBT use, experiences, and treatment response among *Breathe* respondents.

**Methods:**

Enrolled adolescents with self-reported anxiety, aged 13 to 19 years, were randomly allocated to *Breathe* or webpages. Self-reported demographics and anxiety symptoms (Multidimensional Anxiety Scale for Children—2nd edition [MASC-2]) were collected preintervention. Automatically-captured *Breathe* or webpage use and self-reported symptoms and experiences (User Experience Questionnaire for Internet-based Interventions) were collected postintervention. *Breathe* respondents also reported their perceived change in anxiety (Global Rating of Change Scale [GRCS]) following program use. Descriptive statistics summarized usage and experience outcomes, and independent samples *t* tests and correlations examined relationships between them. The MCID was calculated using the mean MASC-2 change score among *Breathe* respondents reporting somewhat better anxiety on the GRCS.

**Results:**

Adolescents were mostly female (382/536, 71.3%), aged 16.6 years (SD 1.7), with very elevated anxiety (mean 92.2, SD 18.1). Intervention use was low for adolescents allocated to *Breathe* (mean 2.2 sessions, SD 2.3; n=258) or webpages (mean 2.1 visits, SD 2.7; n=278), but was higher for *Breathe* (median 6.0, range 1-6; 81/258) and webpage respondents (median 2.0, range 1-9; 148/278). Total user experience was significantly more positive for *Breathe* than webpage respondents (*P*<.001). *Breathe* respondents reported program design and delivery factors that may have challenged (eg, time constraints and program support) or facilitated (eg, demonstration videos, self-management activities) program use. The MCID was a mean MASC-2 change score of 13.8 (SD 18.1). Using the MCID, a positive treatment response was generated for 43% (35/81) of *Breathe* respondents. Treatment response was not correlated with respondents’ experiences or use of *Breathe* (*P*=.32 to *P*=.88).

**Conclusions:**

Respondents reported positive experiences and changes in their anxiety with *Breathe*; however, their reports were not correlated with program use. *Breathe* respondents identified program design and delivery factors that help explain their experiences and use of iCBT and inform program improvements. Future studies can apply our measures to compare user experiences between internet-based interventions, interpret treatment outcomes and improve treatment decision making for adolescents with anxiety.

**Trial Registration:**

ClinicalTrials.gov NCT02970734; https://clinicaltrials.gov/ct2/show/NCT02970734

## Introduction

### Background

Anxiety disorders are the most prevalent mental health concern in children and adolescents, affecting about 8% to 11% of youth [1-3]. Children and adolescents with anxiety disorders are at increased risk of academic and social difficulties and have an increased likelihood of developing secondary anxiety disorders and depression [4,5]. There is strong research evidence supporting the efficacy of cognitive behavior therapy (CBT) as first-line treatment of mild-to-moderate child and adolescent anxiety disorders with number needed to treat ranging from 3 to 6, but also some evidence that CBT is not significantly more effective than active control with support and education materials [6,7]. Understanding options for treatment delivery and for whom it may be best suited is a key area in CBT research, as face-to-face CBT is not always accessible [8], and there are high dropout rates of children and adolescents in traditional outpatient therapy treatment, ranging from 20% to 70% [9].

Internet-based CBT (iCBT), with its self-help format, can increase the access and availability of CBT for adolescents with mild-to-moderate anxiety [10,11]. Recent systematic reviews and meta-analyses demonstrate that in reducing anxiety in adolescents, iCBT has comparable effectiveness with traditional, face-to-face CBT [10,12-14] and is more effective than waiting for treatment [10,13,15-18]. Unlike face-to-face CBT where treatment may involve use of a workbook and in-person meetings with a therapist, iCBT provides therapeutic content and strategies through structured modules and activities (Web-based or offline) that involve the use of multimedia (eg, video and audio) and other technological features (eg, drop-down response menus, animated demonstrations, and interactive quizzes) [19,20]. The use of iCBT can be self-led or therapist guided (synchronous or asynchronous support provided during use), and programs can include varied levels of additional communication, such as reminder emails or follow-up phone calls, to encourage use, troubleshoot issues, or deliver feedback to users during the program.

Evaluations of adolescent experiences with various iCBT program delivery and content formats have revealed *good* program usability (eg, program had few errors and it was easy to learn to use) [21-24], moderate-to-strong credibility (eg, the program contained expert and reliable information), promising treatment expectancy (eg, users’ expressed confidence in the benefits of the program) [21,25-30], and moderate-to-high rates of satisfaction and acceptability (eg, users considered the content relatable and users would recommend the program to others) [26,28,31]. Yet, low usage patterns have been consistently reported in the literature, with typically more than 50% of participants not completing an iCBT program as part of a research study [14,17,32-34]. These discordant outcomes contribute to a lack of clarity about how program usability, credibility, satisfaction, and usage relate to each other as part of an adolescent’s iCBT experience.

Other aspects of the user experience, such as psychosocial barriers and facilitators to program usage, adolescents’ perceived program impacts (eg, perceived effects on health outcomes), and adolescents’ identification of the minimum change in anxiety symptoms that they would accept to make it worth completing an iCBT program (the minimal clinically important difference [MCID] [35]), have not been explored. Yet, these aspects can deepen the understanding of how adolescent users of iCBT perceive programs and experience their use in day-to-day life. Establishing an MCID for the change in anxiety symptoms experienced following a program provides a preferred treatment effect among adolescent users [36]. An adolescent-defined MCID could inform user-centered treatment planning and advance methodological approaches in studies of iCBT effectiveness by framing the estimation of treatment effects [35-37].

### Objectives

We conducted a prospective study of iCBT users’ experiences in the context of a large-scale, parallel design randomized controlled trial (RCT). The large-scale trial was designed to evaluate the effectiveness of an iCBT program developed by our research team, *Being Real, Easing Anxiety: Tools Helping Electronically* (*Breathe*), in reducing anxiety symptoms among adolescents aged 13 to 19 years compared with webpages detailing anxiety resources (resource-based webpages, a usual self-help intervention). Within this trial, we had four distinct objectives for the user experience study: (1) to determine the adolescents’ usage of the *Breathe* program and resource-based webpages, (2) to define the adolescents’ user experiences with the *Breathe* program and the resource-based webpages and examine whether experiences differ between program and webpage use, and (3) to have adolescent users of the *Breathe* program define an MCID for anxiety symptoms after program use, and (4) to explore relationships among the user experiences, program usage, and the MCID among those adolescents who used the *Breathe* program. The overall intent of these objectives was to examine self-reported user experience data and automatically captured program usage data together for a better understanding of the relationship between behavioral (objective usage) and experiential (subjective usage, user experience, and MCID) data [38-40] to explain and understand iCBT outcomes, not to evaluate intervention effectiveness.

## Methods

### Study Design

The RCT was conducted across Canada. We embedded user experience outcome measures (user experience and MCID) and automatically captured intervention data (usage) into pre- and postintervention time points of the trial. The Research Ethics Boards at the University of Alberta approved the trial (ClinicalTrials.gov identifier: NCT02970734; Evaluating an Internet-Based Program for Anxious Adolescents). The trial commenced on November 21, 2016, and the final date of data collection was November 22, 2018.

### Participant Recruitment and Eligibility

Adolescents were recruited for trial participation between November 21, 2016, and July 1, 2018. Recruitment was conducted through the trial’s social media platforms (Facebook, Twitter, Tumblr, and Instagram) with posts and paid advertisements across Canada and through health care professionals who provided study pamphlets to prospective participants seeking mental health care in specialty care clinics, primary care clinics, and schools in Edmonton, Alberta; Hamilton, Ontario; and Halifax, Nova Scotia. Advertisements and pamphlets directed adolescents to view the trial website [41], which provided details on the trial, including eligibility criteria, the screening and enrollment process, information on anxiety, and the research team’s contact information.

Adolescents interested in participation were screened for eligibility using a secure Web-based application, Research Electronic Data Capture (REDCap). Inclusion criteria were as follows: (1) a minimum score of 25 on the Screen for Child Anxiety Related Disorders [42], indicating the presence of clinical anxiety symptoms; (2) the ability to read and write English; (3) regular access to a telephone and a computer system with high-speed internet service; and (4) the ability to use the computer to interact with Web material. Adolescents were ineligible for participation if they (1) screened as high risk for self-harm via four items from the Ask Suicide-Screening Questionnaire [43] (a *yes* answer to thoughts about killing oneself in the past week or a prior attempt), (2) indicated the possible presence of a psychosis-related disorder via the 5-item Schizophrenia Test and Early Psychosis Indicator [44] (an affirmative response to any item), (3) screened positive for harmful or hazardous alcohol consumption via the 3-item Alcohol Use Disorders Identification Test Consumption subscale [45] (a score of ≥3 for females and ≥4 for males), or (4) resided outside of Canada. Ineligible adolescents were provided with suggestions for crisis services and other helplines (ie, Canadian Association for Suicide Prevention and Kids Help Phone) and websites where evidence-based information on alcohol use, psychosis, and self-harm was available.

### Procedures for Informed Consent and Assent

The consent/assent process took place in REDCap. Adolescents were provided an information sheet on the trial and asked several yes/no questions to ensure consent/assent was informed. Those aged 15 to 17 years were able to consent to the study on their own behalf; adolescents aged 13 and 14 years required online parental consent in addition to their assent to participate. Parental consent followed the same Web-based process described for adolescents. Once consent and assent were obtained, adolescents were enrolled in the trial and randomly assigned using a computer-generated sequence with a 1:1 allocation ratio to either the *Breathe* program or the resource-based webpages. This was an open-label trial, and adolescents were notified of their assigned intervention via an email that included instructions for logging into the study website.

### The Breathe Program

**Figure 1:**
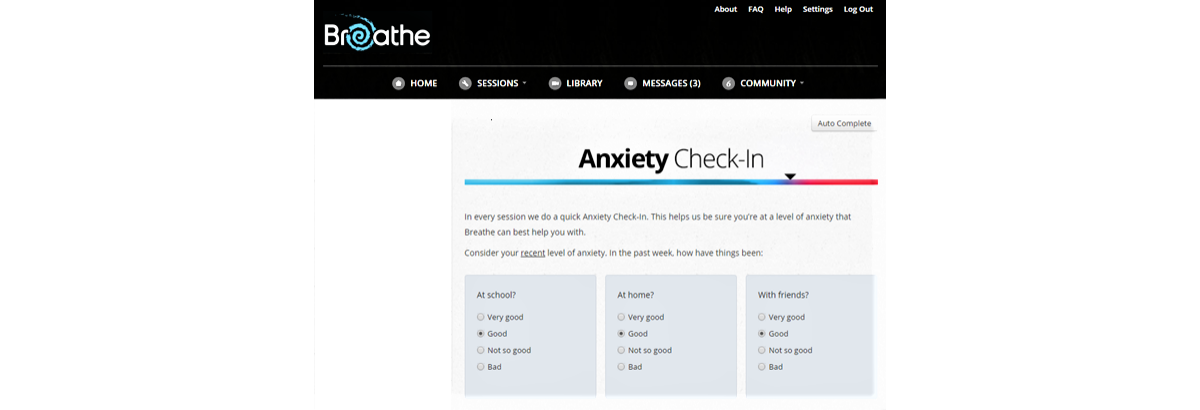
A screenshot of the Check-in activity within the *Breathe* program.

**Figure 2:**
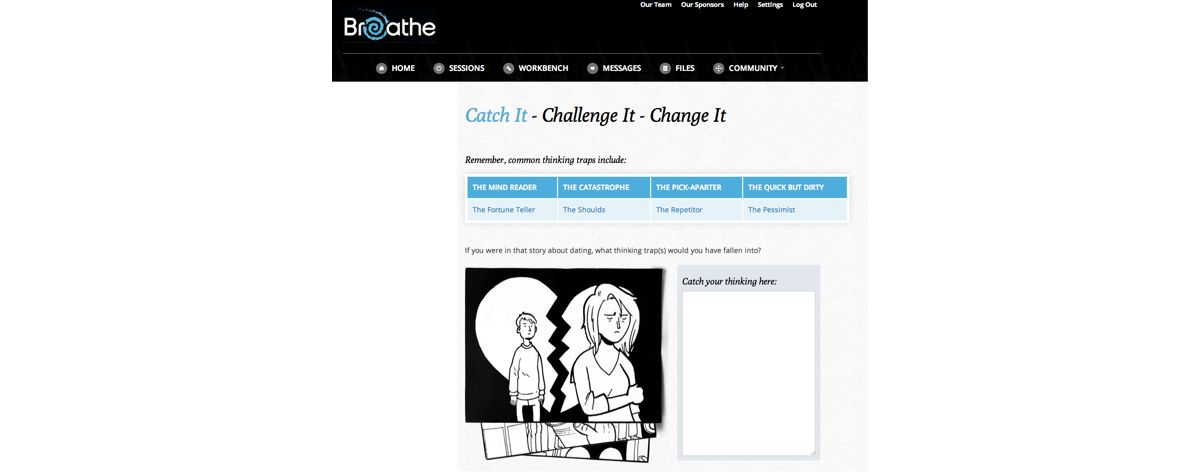
A screenshot of the Discover section within the *Breathe* program.

**Figure 3:**
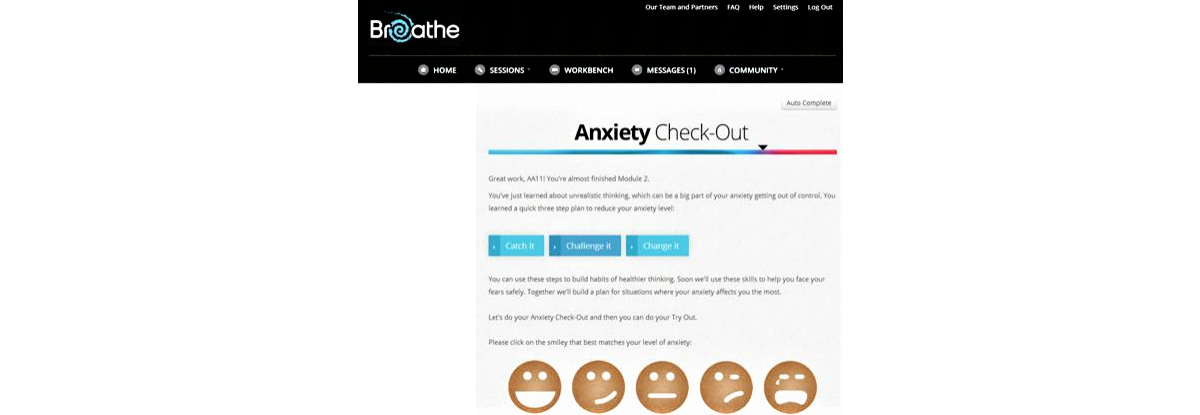
A screenshot of the Check-out activity within the *Breathe* program.

**Figure 4:**
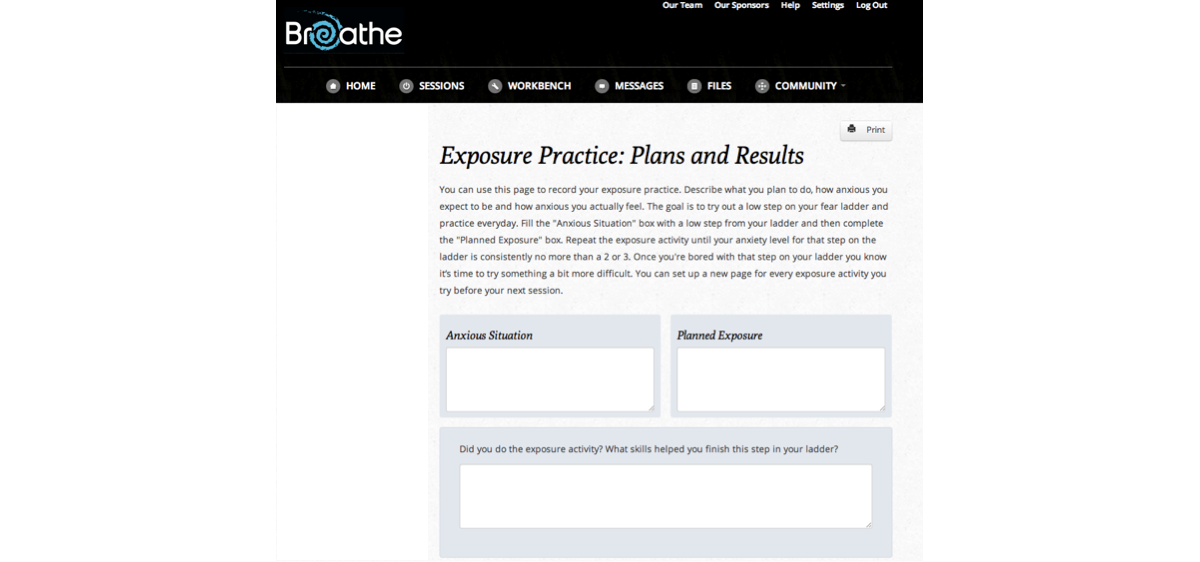
A screenshot of the Try Out activity within the *Breathe* program.

The *Breathe* program for mild-to-moderate anxiety symptoms among adolescents is described in detail elsewhere [46]. In brief, the program was delivered via Intelligent Research and Intervention Software (IRIS), a secure, password-protected website. The program consisted of six iCBT sessions, with each session requiring approximately 30 min to complete; it was suggested that participants complete one session per week in a location convenient for them. Each *Breathe* session included four components: *Check-in*, *Discover*, *Check-out*, and *Try Out*. *Check-in* involved adolescents rating their social-emotional functioning over the past week and indicating whether they had thoughts of self-harm or harming others. Check-in served as a risk management strategy. If a safety issue was flagged (eg, decompensation in anxiety symptoms between sessions and thoughts of self-harm), there was a trigger in IRIS to notify the research assistant to contact the adolescent (and potentially the parent(s) depending on the concern) by phone within 36 hours to assess whether the adolescent required more immediate care and to provide emergent or nonemergency resources. A safety video that included recommendations for immediate safety planning was also provided to adolescents. The *Discover* component of the program introduced the session’s key topics. *Check-out* involved adolescents reflecting on their responses to session content. *Try Out* outlined activities for practicing the session’s key concepts and skills before the next session. An overview of session content is provided in Table 1, and Figures 1-4 provide screenshots of the *Breathe* program.

**Table 1:** An overview of the content presented in the six sessions of the *Breathe* program.

Session	Content covered	Description
1	Psychoeducation	Introduction to the *Breathe* program; psychoeducational information on anxiety and common symptoms (eg, *fight or flight* response and normalization of anxiety); and how cognitive behavioral therapy can be used to treat these symptoms
2	Avoiding avoidance and constructing a fear hierarchy	Identifying avoidant behavior that might be fueling anxiety; strategies for how to avoid avoiding (creating a rewards list); and planning for how to face your worries (*exposure* activities)
3	Relaxation skills	Presentation and practice of common relaxation strategies (eg, deep breathing, visualization, and progressive muscle relaxation)
4	Cognitive distortions	Identifying thinking traps; understanding the *thoughts-feelings-actions* cycle; practice strategies to break out of thinking traps
5	Realistic thinking	Recognizing unrealistic beliefs (eg, perfectionistic and control) and learning strategies for positively reframing them (eg, catch-challenge-change)
6	Fear hierarchy practice, concept integration and relapse prevention	Completing exposure activities; summarizing concepts learned in the *Breathe* program; planning for the future and maintaining gains

Animations, embedded video, audio playback, graphic novel style vignettes, image maps, timed prompts, and on-screen pop-ups were embedded in the program to provide an interactive and multimodal experience. Features based on persuasive systems design [47] were employed to promote program engagement and use: tailoring (provided customized content based on preferences or actions), self-monitoring (progress was tracked and presented virtually to encourage self-reflection), suggestions (key information was provided to help meet users’ goals or needs), and reminders (weekly emails were provided to help users continue with the program and provide notifications of the release of new sessions). Brief Web-based and telephone support was also provided. Participants were assigned a *Breathe* coach, a trained paraprofessional, who initiated an optional telephone coaching session after session 1. The telephone call was not designed as a therapy session but was offered to answer any program-specific questions and to help participants prepare to complete program activities (ie, exposure activities). Participants were not required to complete the call to proceed with the program. Users were also provided with the option for a summary of each session to be emailed to an identified parent or guardian after each completed session.

### Resource-Based Webpages

**Figure 5:**
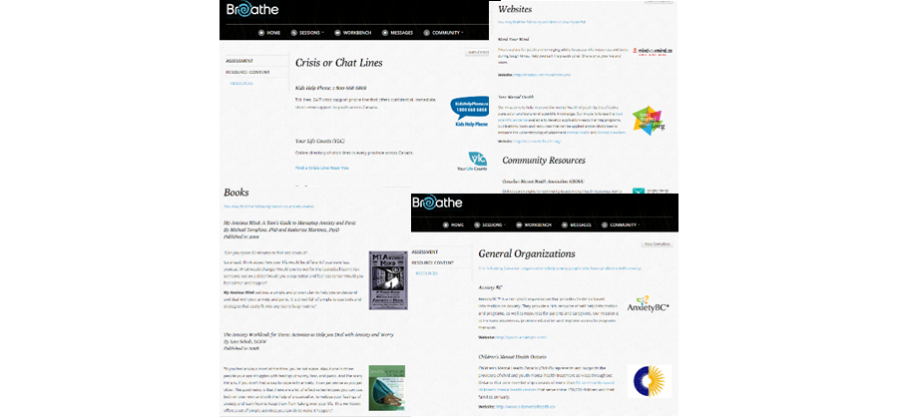
A collage of screenshots from the resource-based webpages.

The resource-based webpages included suggestions of anxiety-based books and educational websites, contact information for local and national crisis lines, and information on the emergency department and other crisis mental health resources. Figure 5 provides a screenshot of the webpages. Webpage users were permitted unlimited access through IRIS over a 6-week period; the same time frame as the *Breathe* program was used. No coaching, safety, or anxiety monitoring was provided during the webpage use.

### Data Collection

We collected user experience data at the preintervention (baseline) and postintervention (6 weeks following enrollment) assessment time points of the trial (Table 2); assessments were independent of an adolescent’s intervention progress or use. Data collection was embedded in IRIS to allow for electronically captured, securely stored, encrypted, and password-protected data. Adolescents who completed outcome measures at the postintervention time point were given a token of appreciation (Can $25 electronic gift card).

**Table 2:** A summary of the study’s assessment time points.

Measure	Time point	
	Preintervention	Postintervention
Demography	X^a^	—^b^
Multidimensional Anxiety Scale for Children	X	X
User Experience Questionnaire for Internet-based Interventions	—	X
Intervention usage	—	X
Global Rating of Change Scale	—	X

^a^X: measure completed.

^b^Not applicable.

### Measures

#### Demography

Adolescent demography included self-reported birth date (used to calculate participant’s age), gender, and province of residence.

#### Multidimensional Anxiety Scale for Children

Anxiety symptoms were reported using the Multidimensional Anxiety Scale for Children—2nd Edition (MASC-2) [48]. The MASC-2 is based on the original MASC [49] that was revised to assess a broader range of anxiety symptoms in children and adolescents aged 8 to 19 years. The MASC-2 is one of the most widely used self-report measures in trials involving adolescents with anxiety because of the brevity of the measure and simplicity of its administration [50]. It consists of 50 items that assess emotional, physical, cognitive, and behavioral symptoms of anxiety using 6 scales and 4 subscales. Adolescents respond using a 4-point Likert scale, ranging from 0 (*never true about me*) to 4 (*often true about me*). The questionnaire yields several scores, including a total raw score and standardized *t* scores based on 18,000 North American children and adolescents aged 8 to 19 years. The scale has acceptable internal consistency (a coefficient alpha of .92 for the self-reported total score), test-retest reliability (all correlations >.80; *P*<.001) [50], and strong convergent validity with other published measures of anxiety symptoms [50].

#### Intervention Usage

We defined intervention usage as adolescent’s use of the *Breathe* program or the resource-based webpages during the 6-week intervention period. Intervention usage was automatically recorded in IRIS using the number of *Breathe* sessions completed per allocated adolescent (a maximum of six sessions) and webpages visited per allocated adolescent (no maximum).

#### User Experience Questionnaire for Internet-Based Interventions

We developed the User Experience Questionnaire for Internet-based Interventions (UEQII) to evaluate and compare adolescents’ self-reported user experience across internet-based interventions (Multimedia Appendix 1). UEQII items were informed by previously published questionnaires and key literature on user experiences [51-53]. Items were tested for face and content validity [54]. The UEQII assesses the user experience through the three constructs: (1) satisfaction and acceptability: global satisfaction, helpfulness, expectations met, convenience, engagement, privacy, and preference for mode of delivery; (2) credibility and impact: confidence in treatment, skill development, and perceived treatment effectiveness; and (3) adherence and usage: ease of use, including technical, psychosocial, and general barriers and facilitators to intervention use.

Adolescents allocated to either the *Breathe* program or resource-based webpage responded to 21 items (*Core* items) on their user experience using a 4-point Likert scale, ranging from 0 (*really worsened* or *not at all*) to 4 (*really improved* or *completely*). An additional 15 items specific to the *Breathe* program experience (items 22-36; *Treatment* items) were completed by adolescents who used the *Breathe* program. If an adolescent responded *not at all* or *slightly* to items 30, 32, or 34, an open text box appeared (subsidiary questions 30a, 32a, and 34a) for the adolescent to elaborate on their experience. Items 35 and 36 were also open text boxes where adolescents could describe what they considered to be the most challenging and enjoyable aspects of the *Breathe* program, respectively. There was not an option for adolescents to skip certain questions.

#### Global Rating of Change Scale

We used a Global Rating of Change Scale (GRCS) that contained a single question with an 11-point Likert scale (ranging from +5 to 0 to −5) to allow *Breathe* program users to indicate the degree to which their anxiety had changed for the better, for the worse, or whether they experienced no change at all as a result of participating in the *Breathe* program. GRCS are widely used in clinical and research settings and are reproducible, clinically relevant, and sensitive to change [55]. To validate the usefulness of the GRCS before calculating the MCID, we calculated the correlation between GRCS scores and pre- and postintervention MASC-2 mean change scores among *Breathe* users. On the GRCS, the smallest change in anxiety symptoms that adolescents identified as important after completing the program [35,56] was used to calculate the MCID.

### Data Analysis

All enrolled participants were included in the analysis of demographic, MASC-2, and intervention usage data; no data imputation strategies were used. For analysis of UEQII and GRCS data, including the MCID calculation, we included adolescents who accessed their assigned intervention at least once during the trial intervention period (ie, those allocated to the *Breathe* program completed at least one session and those allocated to the resource-based webpages visited at least one webpage). This criterion ensured that adolescents commented directly on their experience with the intervention they received. For adolescents who had some missing data among the measures, we used pairwise deletion to maximize the use of all available data on an analysis-by-analysis basis. Normality testing was conducted for all variables. We used means (SDs), median (range), or number (proportion) to describe findings, as appropriate. To compare differences and explore relationships between variables, we conducted independent *t* tests and Pearson correlations (r) for parametric data, and Spearman rank-order correlation coefficients (Spearman rho) and point-biserial correlations for nonparametric data (Pearson product-moment correlation, r_pb_). Data analysis was conducted with IBM SPSS Statistics 25. The significance level was set at *P* less than or equal to .05.

#### Demography

Participant demographics (age, gender, and province of residence) were summarized using means (with SDs) and numbers (proportions).

#### Anxiety Symptoms

The MASC-2 responses were entered in the Multi-Health Systems Online Assessment Center to generate total raw scores and validated *t* scores. We calculated pre- and postintervention symptom scores for each adolescent.

#### Intervention Usage

The mean number (with SD) of completed *Breathe* sessions and webpages visited was calculated at the postintervention time point. Interquartile ranges were used to establish data cutoffs (ie, high-/low-intervention users) to assist with data interpretation. We explored the relationship between intervention usage (the number of completed *Breathe* sessions or webpages visited) and user experience (UEQII total and subscale scores) using Pearson or Spearman correlation.

#### User Experience

User experience data were summarized using means and standard deviations. Multiple construct and total scores were calculated (Multimedia Appendix 2) with higher UEQII scores, indicating a more highly rated (positive) user experience. For both *Breathe* program and resource-based webpage users, we calculated total scores for all *core* user experience items and total subscale scores for each of the three *core* constructs. Among *Breathe* program users, we calculated total scores for all *treatment* user experience items, total subscale scores for each of the three *treatment* constructs, and a total score of all UEQII items by summing the core and treatment items. IQRs were used to establish cutoffs for the scores (ie, first quartile=*low*, second quartile=*moderate*, third quartile=*good*; and fourth quartile=*very good* user experience) to assist with data interpretation; values were rounded up to the nearest whole number for categorization. We tested differences between the user groups for the core all items total score and the three subscale construct total scores using independent samples *t* tests. Open-ended responses from *Breathe* users on the UEQII were extracted verbatim. A basic thematic analysis was conducted by a single author (AR) and reviewed by a second author (AN) [57]. Similar responses were grouped together based on an open, inductive coding process that involved analyzing the explicit content of each response (a semantic approach) [58]. A minimum of two responses were required to generate a theme. Themes are described, and the number of responses per theme are reported.

#### Global Rating of Change

The total and subgroup responses to the GRCS were summarized using means with standard deviations and numbers and proportions. We created 11 subgroups based on adolescents’ responses to the GRCS (a subgroup for each response value on the scale). We also applied the following interpretation to the GRCS scores:

- Adolescents who reported 0 on the GRCS were considered to have experienced no change in their anxiety.
- Adolescents who reported +1 (almost the same, hardly better at all) were considered to have experienced a very small change, but one that may not be clinically relevant.
- Adolescents who reported +2 (somewhat better) on the GRCS were considered to have experienced a small change in their anxiety.
- Adolescents who reported +3 (much better) were considered to have experienced a moderate change in their anxiety.
- Adolescents who reported +4 (a great deal better) or +5 (a very great deal better) were considered to have experienced a large change in their anxiety.

The scores of adolescents who reported a worsening of anxiety symptoms (−1 to −5) were grouped and classified in a similar manner.

#### Minimal Clinically Important Difference

The anchor-based method, the most commonly used method, was used to calculate the MCID. This method involved comparing the change score on the MASC-2 with the GRCS score, which served as the *anchor* [59]. MCID calculation involved three steps. First, we calculated the change in MASC-2 pre- and postintervention total raw scores for each adolescent. Second, we calculated the mean change in the MASC-2 total raw scores for each of the GRCS response subgroups that were created (*no change*, *very small change*, *small change*, *moderate change*, and *large change*). Third, we identified the mean change in MASC-2 scores for adolescents who reported experiencing a *small change* in their anxiety (ie, a +2 response rating on the GRCS, *somewhat better*) to provide the final MCID estimate [35,60,61]. The GRCS response rating used for the MCID estimate (+2) was based on the decision from research team clinicians who care for adolescents with anxiety and have experience using the MASC-2, who felt the +2 estimate (small change) would be relevant to informing their approach to treatment and be considered a positive response in the clinical setting. This GRCS change of 2 points on an 11-point scale is consistent with the MCID (change) of half a standard deviation from a large systematic review of health care outcome studies [62]. In addition to the MCID estimate, the number (proportion) of adolescents who reached (or surpassed) the MCID threshold of a small change in their anxiety improvement was calculated to identify *Breathe* program *treatment responders*. We used point-biserial correlations (a special case of Pearson product-moment correlation, *r*_pb_) to determine the relationship between treatment response (dichotomous variable: treatment responder or nonresponder) and several user experience and usage variables (user experience construct and total scores and the number of *Breathe* sessions completed).

## Results

### Participant Demographics

The total number of adolescents enrolled in the trial was 536 (258 allocated to the *Breathe* program and 278 allocated to the resource-based webpages). Table 3 presents the characteristics of the adolescents before intervention use. The average age of participants was 16.6 years (SD 1.7), and most participants identified themselves as female (382/536, 71.3%). More than two-thirds of adolescents lived in the following 3 Canadian provinces: Ontario (145/536, 27.1%), British Columbia (134/536, 25.0%), and Alberta (81/536, 15.1%). The average baseline MASC-2 total raw score was 92.2 (SD 18.1), with an associated *t* score of 74.9 (SD 9.7; n=408), indicating a *very elevated* level of anxiety.

**Table 3:** Preintervention demographics of enrolled adolescents organized by total adolescents enrolled and total adolescents assigned to each intervention.

Demographic variable		All enrolled adolescents (n=536)	*Breathe* program adolescents (n=258)	Resource-based webpage adolescents (n=278)
**Age (years), mean (SD)^a^**		16.6 (1.7)	16.5 (1.5)	16.7 (1.9)
	No response, n (%)	6 (1.1)	5 (1.9)	1 (0.4)
**Gender, n (%)**				
	Female	382 (71.3)	190 (73.6)	192 (69.1)
	Male	24 (4.5)	13 (5.0)	11 (4.0)
	Other	14 (2.6)	5 (1.9)	9 (3.2)
	No response	116 (21.6)	50 (19.4)	66 (23.7)
**Canadian province of residence, n (%)**				
	Alberta	81 (15.1)	40 (15.5)	41 (14.8)
	British Columbia	134 (25.0)	69 (26.7)	65 (23.4)
	Manitoba	17 (3.2)	9 (3.5)	8 (2.9)
	New Brunswick	8 (1.1)	5 (1.9)	3 (1.1)
	Newfoundland and Labrador	7 (1.3)	4 (1.6)	3 (1.1)
	Northwest Territories	1 (0.2)	1 (0.4)	0 (0.0)
	Nova Scotia	24 (4.5)	10 (3.9)	14 (5.0)
	Ontario	145 (27.1)	68 (26.4)	77 (27.7)
	Prince Edward Island	3 (0.6)	2 (0.8)	1 (0.4)
	No response	116 (21.6)	50 (19.4)	66 (23.7)
**Multidimensional Anxiety Scale for Children—2nd Edition** **(total raw score), mean (SD)**		92.20 (18.1)	92.65 (16.9)	91.77 (19.3)
	No response, n (%)	125 (23.3)	54 (20.9)	71 (25.5)

^a^Adolescents indicated whether they belonged to the 13 to 14 years or 15 to 17 years age category, or neither, as part of eligibility screening. Adolescents were not required to provide their exact age to participate in the study.

### Intervention Usage

Table 4 displays the total number of iCBT sessions completed by adolescents allocated to the *Breathe* program. The average number of iCBT sessions completed by all 258 allocated adolescents to *Breathe* was 2.2 (SD 2.3). Of 258 adolescents, 50 (19.4%) completed the entire six-session program. Using IQRs and the 75th percentile as a cut point, 27.9% (72/258) adolescents completed four or more sessions of the *Breathe* program and were considered to be active *Breathe* participants. Table 5 presents the total number of webpages visited by 278 adolescents allocated to access the anxiety-based resource webpages. The average number of webpages visited by adolescents was 2.1 (SD 2.7). At least one webpage was visited by 196 of 278 (70.5%) adolescents.

**Table 4:** The total number of *Breathe* sessions completed by allocated adolescents.

Total number of *Breathe* sessions completed	Number (proportion) of allocated adolescents (n=258), n (%)
0	91 (35.3)
1	47 (18.2)
2	27 (10.5)
3	21 (8.1)
4	15 (5.8)
5	7 (2.7)
6	50 (19.4)

**Table 5:** The total number of anxiety-based resource webpages visited by allocated adolescents.

Total number of webpages visited	Number (proportion) of allocated adolescents (n=278), n (%)
0	82 (29.5)
1	90 (32.4)
2	31 (11.2)
3	13 (4.7)
4	18 (6.5)
5	9 (3.2)
6	5 (1.8)
7	5 (1.8)
8	2 (0.7)
9	23 (8.3)

### User Experiences

The median number of sessions completed by *Breathe* respondents was 6.0 (range 1-6). Moreover, of 81 *Breathe* respondents 61 (75%) were active participants in the program, with 43 (53.1%) completing the entire program. Among 278 adolescents allocated to the resource webpages, 148 (53.6%) provided postintervention user experience data and visited at least one webpage (herein referred to as webpage respondents). The median number of webpages visited by webpage respondents was 2.0 (range 1-9).

Table 6 presents the responses to user experience questions and differences in experiences between *Breathe* and webpage respondents (score range 0 [not at all] to 4 [completely], with higher scores indicating a more positive rating). Across both interventions, adolescents reported that the information was easy to understand (*Breathe* respondents: mean 3.5, SD 0.7; webpage respondents: mean 2.8, SD 1.2), adolescents trusted the information from the intervention (*Breathe* respondents: mean 3.6, SD 0.7; webpage respondents: mean 3.1, SD 1.0), the internet was a good method for delivering the information (*Breathe* respondents: mean 3.7, SD 0.6; webpage respondents: mean 2.9, SD 1.3), and the intervention was easy to use (*Breathe* respondents: mean 3.3, SD 0.6; webpage respondents: mean 2.4, SD 1.2). *Breathe* and webpage respondents did not consider computer access or availability and internet or technical problems as major barriers to using the interventions. *Breathe* respondents reported that personal (*Breathe* respondents: mean 1.8, SD 1.2; webpage respondents: mean 2.5, SD 1.4) and school (*Breathe* respondents: mean 1.9, SD 1.4; webpage respondents: mean 2.4, SD 1.5) commitments limited their intervention use more so than adolescents who used the webpage (*P* values <.001).

Table 7 presents and compares the total UEQII scores for the core user experience constructs and for all core user experience items (items 1-21) for *Breathe* and webpage respondents. *Breathe* users had significantly higher total satisfaction and acceptability (construct 1), credibility and impact (construct 2), and core items total scores than webpage users. We found that the adherence and usage (construct 3) total score was higher among webpage users compared with *Breathe* respondents, but this difference was not statistically significant.

Table 8 and 9 present *Breathe* respondents’ user experiences with the program (treatment items). The most positive user experiences (higher scores) involved how the *Breathe* program looked, the relevance of the information to the user’s situation, and the likelihood of the program being recommended to others. The lowest rated user experience items were the time required to complete the program, exposure activities (facing your fears), and whether the program helped users meet their treatment goals.

*Breathe* respondents provided open-ended responses for UEQII items 30a, 32a, 34a, 35, and 36. Themes associated with these responses are identified in Table 10 with example responses. Adolescents described nervousness or discomfort around completing (or thinking about completing) the telephone coaching call after session 1, limited time or forgetting to complete the sessions and homework activities (Try Outs), and difficulty in understanding the instructions for planned exposure activities (the worry ladder), including breaking down the anxious situation they wanted to overcome. A major theme surrounding program enjoyment related to respondents learning about anxiety and the new coping strategies or techniques to help them manage their worries.

**Table 6:** The differences in core items of the User Experience Questionnaire for Internet-based Interventions between *Breathe* respondents (n=81) and webpage respondents (n=148).

User experience item	*Breathe* respondents, mean (SD)	Webpage respondents, mean (SD)	Test statistic, *t* test (*df*)	*P* value
1. Was it easy to use?^a^	3.3 (0.6)	2.4 (1.2)	8.1 (222.2)	<.001
2. Was it convenient to use?^a^	3.0 (0.9)	1.8 (1.3)	8.2 (215.5)	<.001
3. Was the information easy to understand?^a^	3.5 (0.7)	2.8 (1.2)	5.8 (222.8)	<.001
4. Was the internet a good method for delivering this information?^a^	3.7 (0.6)	2.9 (1.3)	6.2 (217.5)	<.001
5. Were you eager to use it?^a^	2.9 (0.9)	1.9 (1.3)	6.9 (217.5)	<.001
6. Were you satisfied?^a^	3.0 (0.8)	1.8 (1.3)	8.8 (222.7)	<.001
7. Did it meet your expectations?^a^	3.0 (0.8)	1.7 (1.5)	9.4 (227.0)	<.001
8. Did it keep your interest?^a^	2.7 (1.0)	1.4 (1.3)	8.7 (203.7)	<.001
9. Did you trust the information from it?^a^	3.6 (0.7)	3.1 (1.0)	4.7 (217.8)	<.001
10. Did concerns about your privacy (eg, friends or family knowing about your online activities) affect your use of it?^b^	3.0 (1.1)	3.3 (1.0)	−2.4 (227.0)	<.001
11. Did access or availability of a computer affect your use of it?^b^	3.4 (1.1)	3.4 (1.1)	0.3 (227.0)	.74
12. Did technical computer problems (eg, trouble logging in, clicking to the next page) affect your use of it?^b^	3.6 (0.8)	3.6 (0.9)	−0.4 (227.0)	.74
13. Did internet problems (eg, slow or poor connection) affect your use of it?^a,b^	3.6 (0.7)	3.5 (0.9)	1.0 (208.3)	.34
14. Did personal commitments (eg, family time, extracurricular activities) affect your use of it?^a,b,c^	1.8 (1.2)	2.5 (1.4)	−4.0 (187.8)	<.001
15. Did school commitments (eg, class time, homework) affect your use of it?^b,c^	1.9 (1.4)	2.4 (1.5)	−2.4 (226.0)	.02
16. How likely would you be to come back to it if difficulties with your anxiety continue or return?^a,c^	2.6 (1.1)	1.9 (1.4)	4.0 (202.2)	<.001
17. How did your ability to manage your anxiety change by using it?^a,c^	2.9 (0.5)	2.3 (0.6)	8.1 (195.4)	<.001
18. How did you anxiety with activities at school (eg, speaking up in class and taking a test) change by using it?^a,c^	2.7 (0.6)	2.1 (0.6)	7.8 (163.5)	<.001
19. How did your relationship with friends and peers change by using it?^a,c^	2.5 (0.6)	2.2 (0.6)	3.9 (166.1)	<.001
20. How did your relationships with family members change by using it?^a,c^	2.4 (0.6)	2.1 (0.6)	2.6 (156.0)	.01
21. How did your overall anxiety change by using it?^a,c^	2.8 (0.6)	2.2 (0.8)	6.7 (204.6)	<.001

^a^Equal variances not assumed based on Levene test for equality of variances.

^b^Item is reverse scored so that a higher rating now indicates a more positive experience.

^c^N=147 for this analysis.

**Table 7:** The differences between *Breathe* (n=81) and webpage (n=148) respondents in the construct and core item total scores of the User Experience Questionnaire for Internet-based Interventions.

User experience score	Score range	*Breathe* respondents, mean (SD)	User experience indicator^a^	Webpage respondents, mean (SD)	User experience indicator^a^	Test statistic, *t* test (*df*)	*P* value
Construct 1: satisfaction and acceptability	0-32	25.2 (4.2)	Good	16.6 (7.9)	Moderate	9.2 (227.0)	<.001
Construct 2: credibility and impact	0-24	16.9 (2.2)	Very good	14.0 (3.0)^b^	Moderate	7.7 (226.0)	<.001
Construct 3: adherence and usage	0-28	19.9 (4.2)	Moderate	20.7 (4.4)^b^	Good	−1.4 (226.0)	0.18
All core items	0-84	62.0 (8.2)	Good	51.2 (11.1)^b^	Moderate	7.6 (226.0)	<.001

^a^On the basis of quartiles using all adolescent users (*Breathe* program+webpage users): first quartile=low; second quartile=moderate; third quartile=good; and fourth quartile=very good.

^b^N=147 for this analysis.

**Table 8:** *Breathe* respondents’ ratings (n=81) from the User Experience Questionnaire for Internet-based Interventions.

*Breathe* user experience item	Value, mean (SD)
22. Was it a good fit for you?	2.6 (0.8)
23. Did you like the way it looked?	3.2 (0.9)
24. Did the information relate to you and your situation?	2.8 (1.1)
25. Did it help you meet your treatment goals?	2.3 (1.0)
26. Did the reminder emails affect your use of it?	3.0 (1.2)
27. Did the time required to complete the program affect your use of it?^a^	1.9 (1.2)
28. Did concerns about “facing your fears” affect your use of it?^a^	2.2 (1.3)
29. How likely would you be to recommend it to others?	3.0 (0.8)
30. Were the follow-up emails and telephone calls helpful?^b^	2.7 (1.1)
31. Were the homework (“Try Out”) exercises helpful?^b^	2.4 (1.0)
32. Were the homework (“Try Out”) exercises easy to complete?^b^	2.7 (0.9)
33. Was the worry ladder helpful?^b^	2.4 (1.1)
34. Was the worry ladder easy to complete?^b^	2.4 (1.0)

^a^Item is reverse scored so that a higher rating now indicates a more positive experience.

^b^N=80 for this analysis.

**Table 9:** *Breathe* respondents’ user experiences (n=81) presented by user experience construct, treatment items, and all items total scores from the User Experience Questionnaire for Internet-based Interventions.

User experience score	Total score, mean (SD)	Score range	User experience indicator^a^
Construct 1: satisfaction and acceptability	11.6 (2.6)	0-16	Good
Construct 2: credibility and impact	9.8 (2.8)^b^	0-16	Good
Construct 3: adherence and usage	12.2 (2.9)^b^	0-20	Good
Treatment items	33.5 (6.4)^b^	0-52	Good
All items (core + treatment items)	95.3 (13.5)^b^	0-136	Good

^a^Indicator is based on quartiles of *Breathe* users only: first quartile=low; second quartile=moderate; third quartile=good; fourth quartile=very good.

^b^N=80 for this analysis.

**Table 10:** Themes and responses from open-ended items from the User Experience Questionnaire for Internet-based Interventions.

Open-ended question (number of respondents) and theme (number of responses contributing to each theme)^a^			Example verbatim response
**30a. Why were the follow-up emails and telephone calls not very helpful? (n=10)**			
	Anticipating the telephone coaching call was stressful (n=8)	“I was self motivated so the emails just filled my inbox and the call was uncomfortable.” [user 4992]	
	Emails did not motivate program use (n=4)	“Emails didn’t motivate me, made me want to ignore it even more.” [user 1191]	
	Lack of comfort during the telephone coaching call (n=3)	“I like to do things independently and I find it difficult to interact with strangers.” [user 1447]	
**32a. Why was it a challenge to complete the homework? (n=7)**			
	Lack of time for program workload (n=4)	“Hard to make time and to remember to go back to things everyday.” [user 2930]	
	Forgetting (n=2)	“I’d forget to do them.” [user 107]	
	Feasibility (n=2)	“The boxes were small and it was hard to read all of the text.” [user 1483]	
**34a. Why was it a challenge to complete the worry ladder? (n=12)**			
	Instructions/activities were hard to understand (n=4)	“For me there wasn’t enough instructions for it and I was confused.” [user 2449]	
	Uncertainty in completing (n=3)	“It was difficult coming up with all the steps, i didn't have a creative mind with creative ideas.” [user 1253]	
	Difficulty focusing/articulating worries (n=2)	“I felt my worries were too complex to fit into it.” [user 1825]	
**35. What was the most challenging part of the program? (n=80)**			
	Time management (n=24)	“Trying to complete the tasks on time with my schedule.” [user 894]	
	Preparing for or implementing skills outside of the program (n=23)	“Finding the courage to do exposure activities. Also remembering and putting effort into coping strategies while in an anxious situation.” [user 606]	
	Difficulty working with anxiety concerns (thoughts, feelings, and behaviors) on their own (n=20)	“Facing my fears and organizing my thoughts was a challenge because sometimes I would have to dig deep to find answers.” [user 215]	
	Regular program use (n=18)	“Remembering to participate in the program.” [user 1102]	
	Program format (n=2)	“Reading the format was hard to follow.” [user 1006]	
**36. What was the most enjoyable part of the program? (n=80)**			
	Learning new information and skills (n=31)	“Learning more about what I can do to help myself.” [user 1103]	
	Not feeling alone (n=10)	“I think just knowing that I'm not alone with anxiety. Knowing that other people go through it and some people want to help makes me not feel so alone and helpless.” [user 215]	
	Program activities (n=10)	“I really liked the worry ladder and the surveys.” [user 215]	
	Noticing improvement or impact (n=9)	“Seeing what improvements I may have as well as how this program works.” [user 371]	
	Progress monitoring and feedback activities (n=7)	“I think answering the journals, and keeping track of my anxiety every week from school, family and friends.” [user 1253]	
	Developing insights (n=5)	“Introspection and the ability to actually think about the things I'm doing.” [user 1282]	
	Program format or features (n=5)	“Being able to do it online and not have to talk with anyone face to face.” [user 2209]	
	Positive emotions while working on the program (n=4)	“Finishing the session successfully.” [user 752]	
	Telephone coaching call (n=2)	“My phone call with my coach.” [user 1102]	

^a^Adolescents’ responses may have been coded under more than one theme if there were multiple components (themes) to their response.

### Relationships Between Intervention Usage and User Experience

Table 11 presents the relationships between intervention usage and user experience scores for *Breathe* and webpage respondents. The number of *Breathe* sessions completed was significantly correlated with the adherence and usage construct scores for both the core and treatment items, the total score for all treatment items, and the total score for all user experience items.

**Table 11:** The relationship between intervention usage and the user experience of *Breathe* and webpage respondents.

Items			Total number of *Breathe* sessions (n=81)				Number of webpage visits (n=148)	
			Rho		*P* value	Rho		*P* value
**UEQII^a^ core items (1-21)**								
	Construct 1: satisfaction and acceptability	0.10		.37		0.07		.42
	Construct 2: credibility and impact	0.12		.28		−0.02		.84^b^
	Construct 3: adherence and usage	0.22		.05		0.08		.36^b^
	All core items	0.18		.10		0.07		.42^b^
**UEQII treatment items (22-34)**								
	Construct 1: satisfaction and acceptability	0.15		.17		—^c^		—
	Construct 2: credibility and impact	0.22		.06^d^		—		—
	Construct 3: adherence and usage	0.37		<.00^d^		—		—
	All treatment items	0.33		<.00^d^		—		—
**All UEQII items (1-34)**								
	All core and treatment items	0.30		<.00^d^		—		—

^a^UEQII: User Experience Questionnaire for Internet-based Interventions.

^b^N=147 for this analysis.

^c^Not applicable.

^d^N=80 for this analysis.

### Breathe User Ratings of Changes in Anxiety

Among the 258 *Breathe* respondents, 80 (30.6% of allocated adolescents) reported their change in anxiety using the GRCS (score range −5 to +5, with 0=no change). Among these adolescents, 75% (60/80) reported that their anxiety level improved after they had used the program with an average improvement of 2.3 (somewhat better; SD 0.8). For the 5% (4/80) of adolescents who reported that their anxiety was worse after the program, the average worsening rating was 1.3 (mostly same/hardly worse; SD 0.5). In addition, 20% (16/80) of adolescents reported no change in their anxiety after the program. The mean GRCS response among respondents was 1.7 (SD 1.3). Table 12 presents an overview of the GRCS responses from *Breathe* respondents.

**Table 12:** The change in anxiety levels as reported by *Breathe* respondents using the Global Rating of Change Scale.

Change in anxiety (rating)	Number (proportion) of *Breathe* respondents (n=80), n (%)
A very great deal better (+5)	1 (1)
A great deal better (+4)	3 (4)
Much better (+3)	14 (18)
Somewhat better (+2)	36 (45)
Almost the same, hardly better at all (+1)	6 (8)
No change (0)	16 (20)
Almost the same, hardly worse at all (−1)	3 (4)
Somewhat worse (−2)	1 (1)
Much worse (−3)	0 (0)
A great deal worse (−4)	0 (0)
A very great deal worse (−5)	0 (0)

### Relationships Between the Global Ratings of Anxiety Change, Breathe Program Use, and the Breathe User Experience

We did not find a statistically significant relationship between the number of sessions completed (program use) and *Breathe* respondents’ reported changes in anxiety on the GRCS (rho=0.02; *P*=.83). We found that the GRCS was related to the average user experience, including core total score (r=0.41; *P*<.000), treatment total score (r=0.50; *P*<.000), and the all items total score (r=0.49; *P*<.000).

### Minimal Clinically Important Difference

We found a significant positive correlation between the GRCS scores and the MASC-2 change scores among *Breathe* respondents (r=0.27; *P*=.02), providing face validity for the GRCS to indicate changes in adolescents’ anxiety symptoms [55]. To calculate the MCID, we used the mean change in MASC-2 raw scores among *Breathe* respondents (36/80, 45%) who reported a *somewhat better* change in their anxiety (+2; “small change”) on the GRCS. This mean MASC-2 change score was 13.8 (SD 18.1). Therefore, the MCID for the improvement of adolescents’ anxiety following the *Breathe* program was 13.8 points on the MASC-2. Using this estimate, the number of *Breathe* respondents who reached (or surpassed) the MCID threshold and were considered *treatment responders* was 35 of 81 (43%).

### Relationships Between Treatment Response, Breathe Program Use, and the Breathe User Experience

We found no significant point-biserial correlations (*r*_pb_) between the treatment response (treatment responder or nonresponder) of *Breathe* respondents and (1) the number of sessions completed (*r*_pb_=0.05; *P*=.66), (2) UEQII core total score (*r*_pb_=−0.04; *P*=.76), (3) UEQII treatment total score (*r*_pb_=0.02; *P*=.82), (4) UEQII satisfaction and adherence total score (construct 1; *r*_pb_=−0.03; *P*=.32), (5) UEQII credibility and impact total score (construct 2; *r*_pb_=0.02; *P*=.88), (6) UEQII adherence and usage total score (construct 3; *r*_pb_=0.02; *P*=.88), and (7) UEQII all items total score (*r*_pb_=−0.03; *P*=.82).

## Discussion

### Principal Findings

Interest in the *Breathe* program was high, particularly given that recruitment was primarily through social media and required adolescents to self-identify as wanting help for anxiety. Approximately one-third of the participants in the iCBT intervention completed the postintervention evaluation, and three-fourths of them completed more than half the program. For iCBT programs designed and delivered to adolescents with anxiety, program evaluations should aim to understand how iCBT is experienced by adolescents to further ensure its relevance, use, and impact as a self-help treatment [63-66]. As part of a large-scale evaluation of *Breathe*, an iCBT program for mild-to-moderate anxiety symptoms among adolescents, we used user-reported measures to improve our understanding of adolescents’ use of and experiences with iCBT compared with standard resource-based webpages, and what perceived impact adolescent respondents’ experience following the use of an iCBT program. In the study, we recognized that multiple interacting components influence the user experience [67-69]. By using complementary measures—automatically captured administrative data (eg, session completion data) and self-report of program experience and impact data (quantitative and qualitative)—we described and compared distinct but essential parts of the user experience. As a result, we discovered (1) how iCBT program delivery may influence iCBT use and the user experience, (2) technological features and activities of the program associated with user satisfaction and acceptability, and (3) what adolescents report to be an important change in their anxiety after program use.

### Program Delivery, Internet-Based Cognitive Behavioral Therapy Use, and the User Experience

Similar to previously published studies [70], program use was low among all adolescents allocated to the *Breathe* program. On average, adolescents completed a little more than one-third of the program, and approximately 20% of adolescents completed the entire 6-session program, a completion rate that falls within the range of 5% to 50% reported by other studies of iCBT programs [70]. Program use was higher among *Breathe* respondents (ie, approximately one-third of allocated adolescents who provided user experience data), 75% of whom were considered active program participants. This more engaged user group of *Breathe* respondents can be used to explore ways that we might increase program use among other adolescent iCBT users. Although other studies have looked to user demographics to provide explanations in low program use, explanations have been mixed [13,15,18], which suggests new approaches to understanding program use are needed.

Consistent with the literature, *Breathe* respondents described difficulty remembering to work on the program [29,52], concerns with privacy and stigma (eg, others knowing about or judging their help seeking) [30,71,72], time constraints, and conflicting commitments [31,73-75], and delaying or avoiding tasks they found challenging [76,77] as the biggest obstacles to program adherence and use. The time of day when adolescents opted to access the program (ie, immediately after school and before bed) or the portability of the medium used to access it (ie, desktop computer and mobile phone app) could be related to these perceived barriers and require exploration in future studies. A recent review of iCBT programs for children and adolescents with anxiety found that all programs that have undergone empirical testing included some form of program support (eg, teacher administration, weekly therapist emails, and parent-directed modules) [70] so that programs were not solely self-administered and unsupported. Most previously studied iCBT programs with completion rates greater than 50% involved regular therapist or parent involvement to support program use [26,29,78-82]. It may be that this type of support as well as the degree of support provided may help adolescents manage their time and complete challenging program activities [27,81,83-85]. There is a trend in the literature that some type of program support can increase program use or effectiveness of iCBT for children and adolescents [10]; however, inconsistent evidence is published [16,28,86,87], and what type of support, such as when it should be provided and by whom, that improves outcomes is unclear [13,15,17,88]. As part of the *Breathe* program, adolescents received one telephone-based coaching call after completing their first session to prepare adolescents for the skills-based program activities to follow, including exposure activities, that would begin in session 2. Almost half of the adolescents allocated to the *Breathe* program did not go on to complete the next program session and the personalized exposure activities they had set up in session 1 (ie, a hierarchy of activities specific to their worries and fears). Although some adolescents described the call as a positive experience, others considered it *stressful* because they did not know the coach, and some adolescents described avoiding and delaying the call. This mixed response to coach involvement suggests that *how* support is provided is a key aspect of program delivery and the user experience. Some studies of Web-based interventions have described including rapport building activities (eg, introductory telephone call) between adolescents and the adjunct support person before treatment material is discussed (eg, preparing for exposure exercises) [29,84]. Including an activity similar to this may have helped some adolescents begin the *Breathe* program or ameliorate some of the discomfort or nervousness they experienced leading up to or during the coaching call, thereby retaining active participants in the program.

It is important to note that the stage of the program at which user experiences are measured may provide more or less information on the relationship between adolescents’ use of, experiences with, or perceived impact of a program. In this study, we administered our user experience measures after program use. However, moving forward in the field, there is value in formative evaluation during program use. Such evaluations may reveal how the user experience changes over time, how it can be optimized [89], and how to improve the accuracy of collected data on the user experience (eg, reduce recall bias and link user experience domains to specific program sessions). For example, repeated measurement, using log data or routine monitoring of points of program stoppage among adolescents, may help to identify the relationship between program continuation or discontinuation, adolescents’ anxiety states, or program content or features. Use of factor analysis [90] or multiple regression [91] could help to illuminate how different constructs of user experience relate to one another and to intervention use and how the constructs change over the course of treatment.

### Program Features and Activities and the User Experience

Overall, in this study, user experiences were significantly more positive for *Breathe* respondents than for resource-based webpage respondents. The only user experience questionnaire construct for which we found no difference between the two intervention groups was the adherence and usage construct—both the *Breathe* program and webpage respondents reported few concerns with technology or internet accessibility or functionality during the study. Similar to other iCBT studies, *Breathe* respondents reported that the program was easy to understand [92], met their needs [79], and that they were satisfied overall [29,93,94]. Nearly half of the respondents stated that the most enjoyable parts of the program were learning about anxiety, developing new coping strategies, and feeling like others could relate to their situation or worries and vice versa. However, *Breathe* respondents’ satisfaction and acceptability with the program were not correlated with their use of it, suggesting that other program factors need to be explored for their association with iCBT use. A distinguishing feature of *Breathe* compared with the resource webpages was that *Breathe* incorporated instruction and interaction (providing opportunities for *doing*) in addition to information (providing opportunities for *knowing*) as part of the intervention, helping adolescents develop their capacity and competency for self-management rather than redirecting them to alternative resources. *Breathe* respondents liked activities that improved their ability to self-manage their anxiety by informing them, empowering them, or normalizing their experiences. Respondents reported the greatest interest in developing skills that were relatively easier to learn and had a timelier impact (eg, *deep breathing exercises* and *watching videos of other teens with anxiety and relating to them*). When designing an iCBT program, it may be helpful to consider *balancing* the variety and sequence of program content and activities included according to their expected level of effort from the user and the immediacy of benefit. *Breathe* respondents reported positive experiences with more immediate (eg, relaxation or mindfulness techniques) and short-term relief tasks (eg, psychoeducation, normalization, and affirmation of support), suggesting that when long-term relief tasks (eg, exposure activities and homework) are presented in sessions, some immediate and short-term relief tasks should also be included (eg, revisited or presented) to maintain adolescents’ interest and sense of self-mastery or achievement with the program. Combining immediate and short-term relief tasks with long-term ones could potentially offset the discomfort and effort required to persist through more demanding tasks (ie, exposure), making it easier for adolescents to continue with the program.

In addition to program content and activities, technological features are also inherent aspects of iCBT. The *Breathe* program was developed using persuasive systems design components (technology-based interventions designed to reinforce, change, or shape attitudes or behaviors [74]) to increase program engagement, use, and effectiveness. Yet, on average, program use was still low for all allocated adolescents. Persuasive design features are embedded within the program itself, making use of the program a prerequisite for adolescents to experience these features and their *persuasive effects*. The majority of *Breathe* adolescents did not access the first session and were not exposed to such features. Among the adolescents who did use the *Breathe* program, they described specific persuasive design features to be among the most enjoyable features of the program. These features included interactive surveys and graphs (designed to provide feedback, increase adolescents’ awareness of their changes over time, and help with goal setting [95-97]), and video clips showing in-vivo exposure and diaphragmatic breathing (designed to provide step-by-step peer simulations of therapeutic activities [70]). On the basis of adolescent feedback in this study, it may be that the design features did have a positive influence on program use as intended. However, what remains an important question is how to promote adolescents’ initial engagement with a persuasive systems design–based program so that they can experience the program’s features. One strategy may involve the use of preintervention activities, such as readying adolescents for the iCBT program, or assessing the fit between adolescents and the program to improve program initiation and use. For example, a *preview* of an iCBT program could be provided to adolescents before eligibility screening to pique their interest in the program. Incorporating an iCBT program preview could promote a user-centered, decision-making treatment process (adolescents can self-select programs that meet their needs and preferences), streamline the recruitment and eligibility screening process (identifying adolescents who may be unlikely to use the program early on and saving time and resources by redirecting them to treatment alternatives), uphold research or clinical practice ethics (adolescents can avoid a treatment that may be unusable, ineffective, or potentially harmful to them), and stimulate or *kick start* adolescents use of the program (adolescents become intrigued and interested in commencing the program). Another strategy to promote initial program engagement is to incorporate an assessment of beliefs and attitudes before program use. Persuasive technology aims to reinforce, change, or shape users’ attitudes or behaviors toward their health goal [47,98], suggesting that a clear understanding of adolescents’ psychology precedes the selection and use of an intervention. Assessing adolescents’ existing health beliefs and attitudes (eg, treatment expectations, health and technology literacy, and self-efficacy) and treatment goals (eg, desired change in knowledge, skills, or symptoms) preintervention may help determine (1) the potential for successful *persuasion* to occur (an attitude or behavior change) with the use of the iCBT program; and (2) if a positive potential exists, what persuasive system design components may be most appropriate to match the beliefs and goals of the adolescent. Being able to assess and appropriately tailor a program’s persuasive features based on adolescents’ beliefs, attitudes, and goals could improve adolescents’ experience and use of iCBT.

Considering that multiple iCBT components work together to form a complex intervention [99], we recommend connecting the persuasive system design features known to relate to a positive user experience (program reminders, progress and feedback tools, multimedia demonstrations, and flexible program support) with proposed mechanisms of change (CBT content [psychoeducation, skills training], attitude or behavior change processes [techniques that target adolescents’ motivation and sense of mastery]) [70]. Future studies that systematically test the relationship between iCBT features, behavior change processes, user experience, and health outcomes would help to develop working models of iCBT effectiveness. Standardized interviews and patient-reported measures (eg, Ratings of Perceived Helpfulness in Behavior Change [74,100]) may also help researchers determine how iCBT program features have or have not engaged adolescents in behavior change, the reliability of adolescents’ self-awareness/reports on their fit with a program and adolescent to determine the self-reports, and what features were most effective for improving program use.

### Changes in Adolescents’ Anxiety Following Internet-Based Cognitive Behavioral Therapy Use

Previous iCBT studies have measured whether program participation was perceived as *effective or useful* by adolescents [81,92] but have not formally measured the degree of meaningful change in anxiety as experienced by users of a program. This study is the first to quantify a user-reported improvement to an MCID for anxiety symptoms, a common primary outcome of trials to date. Establishing this MCID is an important step in informing future sample sizes for trials of iCBT effectiveness (eg, can provide a clinically meaningful effect size) and interpreting adolescent outcomes (eg, presenting results with a clear meaning behind anxiety changes and implications, such as whether an adolescent is a positive responder to iCBT). Reporting whether changes in anxiety across different programs met an MCID can also assist adolescents, parents, and clinicians in deciding which program best matches their expected treatment response [37,101].

In this study, most adolescents reported that their anxiety was *better* after using the *Breathe* program. On the basis of the MCID estimate generated from adolescents’ ratings, 43% (35/81) of *Breathe* respondents were positive treatment responders. Previous iCBT studies have used clinical severity ratings (ratings have ranged from 0=none to 8=extremely severe) as a proximal indicator of treatment response [27,29,79,81]. However, a clinician has assigned these ratings. For programs used outside a research or clinical setting, the use of an MCID to determine treatment response can reduce costs and time associated with clinician involvement and better reflects the experience of the youth.

For *Breathe* respondents, we did not find a statistically significant relationship between treatment response and the number of program sessions completed. There is mixed evidence as to whether a causal relationship between iCBT use and change in anxiety (a *dose-response* relationship) exists—some studies have found evidence for this relationship [102,103], whereas others have not [104,105]; however, there is consensus that some degree of program use is required to reduce users’ symptoms [106-108]. In our study, adolescents may have discontinued their use of a program (temporarily or definitively) once they felt their symptoms had improved, regardless of their progress in the program. Perceived impact may also be based on unique individual factors, such as treatment expectancy, preintervention anxiety severity, self-regulation abilities, or motivational factors [69,102,109], factors that we did not assess. The lack of association between treatment response and program use further emphasizes the importance of incorporating adolescents’ perspectives in the evaluation of iCBT because commonly used methods (eg, standardized symptom questionnaires) may not fully capture the health and social benefits adolescents want or need from an iCBT program. More research is required to determine what treatment outcomes are important to adolescents who seek to use iCBT apart from those that researchers and clinicians typically administer.

### Strengths and Limitations

This study has several strengths related to the assessment of user experiences of an iCBT program for adolescents with anxiety. Currently, there is considerable heterogeneity in how the user experience is defined and evaluated, with most research being conducted with adult populations [65,69,110,111]. To target our anticipated participants, we used current, key literature [30,52,53,70,112-114] to develop the UEQII. This self-report measure includes three major user experience constructs (construct 1: satisfaction and acceptability, construct 2: credibility and impact, and construct 3: adherence and usage). Each construct provided diverse information to understand the adolescent experiences with an iCBT program as well as our comparison intervention. With the growing number of RCTs evaluating iCBT programs using a technology-based intervention as a control, a method to compare the user experience between two internet-based interventions for adolescents is becoming increasingly important. Although this measure is subject to response bias (recall or social desirability) and relies on adolescents’ insights of their own behaviors or attitudes (experiential data), it provides information that is not directly observable and cannot be captured by traditional diagnostic assessments, a proxy respondent (ie, parent), or digital log data (objective data). In the future, other researchers can use the UEQII by administering the *core* items to other internet-based interventions and adapting the *treatment* items for their intervention under study to narrow in on what specific intervention components meet the needs and preferences of their target users. As a first step before broader use, we recommend that the UEQII undergo further psychometric testing to assess its feasibility and transferability in other contexts, ages, and patient groups and iCBT programs.

This study also has several limitations. First, we used adolescent ratings on a global rating scale (in our case, a GRCS) to calculate the MCID. There is no standard for how to calculate the MCID; therefore, a variety of methods exist and can be used depending on the study sample and data collected (for a review of the different methods, refer to the studies by Copay et al [59], Wells et al [115], Beaton et al [116], and Ebrahim et al [117]). In this study, the anchor-based approach was considered optimal because it maintains the user’s perspective [117-119], an essential perspective with a primarily self-led intervention for an internalizing disorder. However, it is unclear how factors such as treatment preferences, engagement, or expectations may influence individual ratings, and therefore the MCID score (based on an average of individual scores). The GRCS significantly correlated with the MASC-2 change scores, considered a *gold standard* screen of adolescent-reported anxiety symptoms, providing support for the validity of the MCID estimate. Disadvantages of the anchor-based method, however, include the selection of the anchor itself (ie, GRCS) and the potentially arbitrary nature of the MCID cut point for a small change in anxiety (ie, *somewhat better*), although the GRCS change is consistent from other studies [62]. Thus, the MCID estimate calculated can vary between samples with different participant characteristics (eg, baseline severity and previous treatment experiences) [55,59,118]. Moving forward, we recommend that MCIDs be calculated using the same measures (GRCS and MASC-2) for adolescent users of other iCBT programs. A composite MCID estimate can then be generated by amalgamating MCID data across multiple studies to increase the generalizability and validity of the estimate [120] or provide a range of critical MCID values can be provided. The composite and ranges can be corroborated using Delphi (eg, clinical or expert opinion) or distribution-based methods (eg, effect size and standard error of measurement) [59,116], triangulating multiple approaches to calculating the MCID to improve the robustness of the estimate [101].

Finally, in this study, there was a large rate of attrition, which resulted in only about one-third of enrolled adolescents included in the user experience analysis. Attrition is said to be a fundamental characteristic and methodological limitation of longitudinal iCBT studies [121-123]; however, our attrition rates are consistent with dropouts in outpatient therapy settings [9]. Participants in this study reported high levels of anxiety on a standard screening tool (MASC-2, *very elevated*) at preintervention, which reflects a greater severity of anxiety symptoms in those seeking help than those in most minimally supported iCBT studies. This study was inclusive of youth at any stage in their treatment journey, and it is possible that some youth were exploring multiple options to access help and that an iCBT program was not the option of best fit at that time. It is also possible that the limits in timing of the evaluation at baseline and 6 weeks from enrollment may also have impacted the number of respondents as some adolescents may have been excluded who would have engaged further with a longer time course. Thus, our user experience findings may be based on adolescents who are different from those who dropped out of the study. *Breathe* respondents who used the program and completed the postintervention assessments may have had a preference for self-help programs, greater motivation, or commitment to treatment or viewed the program to be highly relevant or beneficial to them [74,121,124]. As the perceptions of adolescents who dropped out were not captured by our evaluation, we are limited in understanding of why an iCBT program is unlikely to be used once accessed. Additional adolescent demographic (eg, urban or rural residence) or clinical information (eg, psychological comorbidities) could help explain the differences in attrition between respondents and nonrespondents or be used to explore mediators or moderators of study participation, but these data were not collected as part of this study. Sample characteristics, such as most adolescents identifying as female, may limit the generalizability of our findings to other adolescents who seek self-help, technology-based interventions to manage their anxiety.

### Conclusions

Given the high prevalence of anxiety disorders, the challenges in accessing CBT, and the interest of young people in internet interventions, iCBT is an important area of clinical research. In this study, we used user-reported measures, including a new measure, the UEQII, to examine the multiple components that influence anxious adolescents’ experiences with an iCBT program compared with that of resource-based webpages. How iCBT is delivered may influence and help explain the relatively low number of session use, perception of time constraints, and other commonly reported challenges to completing a program. The more positive experience that *Breathe* respondents reported compared with webpage respondents may be attributed to the interactive technological features and program activities (eg, graphs, video demonstrations, and learning about anxiety) with specific focus on anxiety-coping skills that were incorporated into the iCBT program. Although most adolescent respondents experienced benefit from an iCBT program, the relationship between adolescents’ use, their experiences, and perceived impact on anxiety is still unclear, indicating that further understanding of what adolescents find challenging and enjoyable about iCBT as well as the characteristics of those who would most benefit from this delivery mode is necessary to optimize its delivery. Future studies can validate the UEQII, test and integrate our program suggestions, and apply our user experience measures toward creating robust treatment planning guidelines, including mechanisms to engage more youth in treatment completion.

## Appendix

Multimedia Appendix 1 The User Experience Questionnaire for Internet-based Interventions.

Multimedia Appendix 2 Scoring of the User Experience Questionnaire for Internet-based Interventions.

Multimedia Appendix 3 CONSORT-EHEALTH checklist (V 1.6.1).

## Abbreviations

Breathe: Being Real, Easing Anxiety: Tools Helping Electronically
CBT: cognitive behavioral therapy
GRCS: Global Rating of Change Scale
iCBT: internet-based cognitive behavioral therapy
IRIS: Intelligent Research and Intervention Software
MASC-2: Multidimensional Anxiety Scale for Children—2nd Edition
MCID: minimal clinically important difference
RCT: randomized controlled trial
REDCap: Research Electronic Data Capture
UEQII: User Experience Questionnaire for Internet-based Interventions
