# Supplementary material for: Examining the Usage, User Experience, and Perceived Impact of an Internet-Based Cognitive Behavioral Therapy Program for Adolescents With Anxiety: Randomized Controlled Trial
Source: JMIR Ment Health. 2020 Feb 5;7(2):e15795. doi: 10.2196/15795 (PMC7055748; doi:10.2196/15795)
Supplement: Multimedia Appendix 1 [file mental_v7i2e15795_app1.pdf]

Multimedia Appendix 1. The User Experience Questionnaire for Internet-based Interventions (UEQII).

Please think about your experience with the [*Breathe* program/ anxiety resources webpage] intervention and rate the following:

1.	Was it easy to use?	Not at all	Slightly	Somewhat	Mostly	Completely
		0	1	2	3	4
2.	Was it convenient to use?	Not at all	Slightly	Somewhat	Mostly	Completely
		0	1	2	3	4
3.	Was the information easy to understand?	Not at all	Slightly	Somewhat	Mostly	Completely
		0	1	2	3	4
4.	Was the Internet a good method for delivering this information?	Not at all	Slightly	Somewhat	Mostly	Completely
		0	1	2	3	4
5.	Were you eager to use it?	Not at all	Slightly	Somewhat	Mostly	Completely
		0	1	2	3	4
6.	Were you satisfied?	Not at all	Slightly	Somewhat	Mostly	Completely
		0	1	2	3	4
7.	Did it meet your expectations?	Not at all	Slightly	Somewhat	Mostly	Completely
		0	1	2	3	4
8.	Did it keep your interest?	Not at all	Slightly	Somewhat	Mostly	Completely
		0	1	2	3	4
9.	Did you trust the information from it?	Not at all	Slightly	Somewhat	Mostly	Completely
		0	1	2	3	4
10.	Did concerns about your privacy (e.g., friends or family knowing about your online activities) affect your use of it?	Not at all	Slightly	Somewhat	Mostly	Completely
		4	3	2	1	0
11.	Did access or availability of a computer affect your use of it?	Not at all	Slightly	Somewhat	Mostly	Completely
		4	3	2	1	0
12.	Did technical computer problems (e.g., trouble logging in, clicking to the next page) affect your use of it?	Not at all	Slightly	Somewhat	Mostly	Completely
		4	3	2	1	0

13.	Did Internet problems (e.g., slow or poor connection) affect your use of it?	Not at all	Slightly	Somewhat	Mostly	Completely
		4	3	2	1	0
14.	Did personal commitments (e.g., family time, extracurricular activities) affect your use of it?	Not at all	Slightly	Somewhat	Mostly	Completely
		4	3	2	1	0
15.	Did school commitments (e.g., class time, homework) affect your use of it?	Not at all	Slightly	Somewhat	Mostly	Completely
		4	3	2	1	0
16.	How likely would you be to come back to it if difficulties with your anxiety continue or return?	Not at all	Slightly	Somewhat	Mostly	Completely
		0	1	2	3	4
17.	How did your ability to manage your anxiety change by using it?	Really Worsened	Somewhat Worsened	No Change	Somewhat Improved	Really Improved
		0	1	2	3	4
18.	How did your anxiety with activities at school (e.g., speaking up in class, taking a test) change by using it?	Really Worsened	Somewhat Worsened	No Change	Somewhat Improved	Really Improved
		0	1	2	3	4
19.	How did your relationships with friends and peers change by using it?	Really Worsened	Somewhat Worsened	No Change	Somewhat Improved	Really Improved
		0	1	2	3	4
20.	How did your relationships with family members change by using it?	Really Worsened	Somewhat Worsened	No Change	Somewhat Improved	Really Improved
		0	1	2	3	4
21.	How did your overall anxiety change by using it?	Really Worsened	Somewhat Worsened	No Change	Somewhat Improved	Really Improved
		0	1	2	3	4
22.	Was it a good fit for you?	Not at all	Slightly	Somewhat	Mostly	Completely
		0	1	2	3	4
23.	Did you like the way it looked?	Not at all	Slightly	Somewhat	Mostly	Completely
		0	1	2	3	4
24.	Did the information relate to you and your situation?	Not at all	Slightly	Somewhat	Mostly	Completely
		0	1	2	3	4

25.	Did it help you to meet your Treatment goals?	Not at all	Slightly	Somewhat	Mostly	Completely
		0	1	2	3	4
26.	Did the reminder emails affect your use of it?	Not at all	Slightly	Somewhat	Mostly	Completely
		0	1	2	3	4
27.	Did the time required to complete the program affect your use of it?	Not at all	Slightly	Somewhat	Mostly	Completely
		4	3	2	1	0
28.	Did concerns about 'facing your fears' affect your use of it?	Not at all	Slightly	Somewhat	Mostly	Completely
		4	3	2	1	0
29.	How likely would you be to recommend it to others?	Not at all	Slightly	Somewhat	Mostly	Completely
		0	1	2	3	4
30.	Were the follow-up emails and telephone calls helpful?	Not at all	Slightly	Somewhat	Mostly	Completely
		0	1	2	3	4
30a.	If you answered 'Not at all' or 'Slightly' to the question above, why were the emails and telephone calls not very helpful?	Open text box				
31.	Were the homework ('Try Out') exercises helpful?	Not at all	Slightly	Somewhat	Mostly	Completely
		0	1	2	3	4
32.	Were the homework ('Try Out') exercises easy to complete?	Not at all	Slightly	Somewhat	Mostly	Completely
		0	1	2	3	4
32a.	If you answered 'Not at all' or 'Slightly' to the question above, why was it a challenge to complete the homework?	Open text box				
33.	Was the worry ladder helpful?	Not at all	Slightly	Somewhat	Mostly	Completely
		0	1	2	3	4
34.	Was the worry ladder easy to complete?	Not at all	Slightly	Somewhat	Mostly	Completely
		0	1	2	3	4
34a.	If you answered 'Not at all' or 'Slightly' to the question above, why was it a challenge to complete the worry ladder?	Open text box				

35.	What was the most challenging part of the program?	Open text box
36.	What was the most enjoyable part of the program?	Open text box
