# Supplementary material for: Examining the Usage, User Experience, and Perceived Impact of an Internet-Based Cognitive Behavioral Therapy Program for Adolescents With Anxiety: Randomized Controlled Trial
Source: JMIR Ment Health. 2020 Feb 5;7(2):e15795. doi: 10.2196/15795 (PMC7055748; doi:10.2196/15795)
Supplement: Multimedia Appendix 2 [file mental_v7i2e15795_app2.pdf]

Multimedia Appendix 2. Scoring of the User Experience Questionnaire for Internet-based Interventions (UEQII).

**Questionnaire for the Control and Treatment groups (Items 1-21):**				
		**Items**	**Reverse Scored Items**	**Total Score Range**
‘Core’ items total score		1-21	10-15	0-84
Construct 1 total score: Satisfaction and Acceptability		1-8	None	0-32
Construct 2 total score: Credibility and Impact		9, 17-21	None	0-24
Construct 3 total score: Adherence and Usage		10-16	10-15	0-28
**Questionnaire for the Treatment group only (Items 22-36):**				
	**Open-field Responses**	**Items**	**Reverse Scored Items**	**Total Score Range**
‘Treatment’ items total score	N/A	22-34	27, 28	0-52
Construct 1 total score: Satisfaction and Acceptability	35, 36	22-24, 29	None	0-16
Construct 2 total score: Credibility and Impact	30a	25, 30, 31, 33	None	0-16
Construct 3 total score: Adherence and Usage	32a, 34a	26, 27, 28, 32, 34	27, 28	0-20
